# Lack of Congruence in Species Diversity Indices and Community Structures of Planktonic Groups Based on Local Environmental Factors

**DOI:** 10.1371/journal.pone.0069594

**Published:** 2013-07-25

**Authors:** Hideyuki Doi, Kwang-Hyeon Chang, Yuichiro Nishibe, Hiroyuki Imai, Shin-ichi Nakano

**Affiliations:** 1 LAFWEDY, Faculty of Agriculture, Ehime University, Matsuyama, Japan; 2 Institute for Sustainable Sciences and Development, Hiroshima University, Higashi-Hiroshima, Japan; 3 Center for Marine Environmental Studies, Ehime University, Matsuyama, Japan; 4 Department of Environmental Science and Engineering, Kyung Hee University, Seocheon-dong, Giheung-gu, Yongin-si, Korea; 5 Atmosphere and Ocean Research Institute, The University of Tokyo, Kashiwa, Japan; 6 Center for Ecological Research, Kyoto University, Otsu, Japan; University of Florida, United States of America

## Abstract

The importance of analyzing the determinants of biodiversity and community composition by using multiple trophic levels is well recognized; however, relevant data are lacking. In the present study, we investigated variations in species diversity indices and community structures of the plankton taxonomic groups–zooplankton, rotifers, ciliates, and phytoplankton–under a range of local environmental factors in pond ecosystems. For each planktonic group, we estimated the species diversity index by using linear models and analyzed the community structure by using canonical correspondence analysis. We showed that the species diversity indices and community structures varied among the planktonic groups and according to local environmental factors. The observed lack of congruence among the planktonic groups may have been caused by niche competition between groups with similar trophic guilds or by weak trophic interactions. Our findings highlight the difficulty of predicting total biodiversity within a system, based upon a single taxonomic group. Thus, to conserve the biodiversity of an ecosystem, it is crucial to consider variations in species diversity indices and community structures of different taxonomic groups, under a range of local conditions.

## Introduction

Elucidation of the factors that control species diversity and community composition in local habitats is essential for protecting biodiversity in small habitats. Many relevant studies have been conducted [1], particularly regarding the development of efficient monitoring methods and conservation practices [2], [3]. Some studies have suggested that environmental heterogeneity [4] and ecosystem size [5] are crucial factors determining diversity and community structure in local habitats. In addition, species richness is believed first to increase, and then to decrease according to the productivity of an ecosystem, thereby producing a hump-shaped relationship [3], [6], [7]. These findings are important when predicting community composition.

The factors that determine local community structures in natural systems are complicated by the range of processes and interactions occurring across trophic guilds [8]. To understand the conservation of a whole community within an ecosystem, these interactions must be considered. However, previous empirical and theoretical studies have mainly focused on restricted numbers of organism groups, in order to reduce the complexity of the relationships with biotic and abiotic factors, and also to simplify interpretation of the results [4].

Similarities among different community assemblages at the same location may facilitate ecological understanding and yield new insights, by highlighting similarities and differences regarding the way in which different assemblages respond to the environment [9], [10]. Nevertheless, demonstrating congruence among responses of community assemblages to environmental factors is complicated. In the present study, we hypothesized that taxonomic groups with similar trophic guilds would show similar species diversity indices and community assemblages according to environmental factors. Further, because similar taxonomic groups have similar life cycles and body sizes [1], we proposed that pairs of taxonomic groups with similar trophic guilds would show greater congruence of species diversity indices and community assemblages.

Lakes and ponds are clearly defined ecological entities in the landscape, and therefore provide good systems for studying biodiversity in relation to environmental gradients [2], [7]. Moreover, within these ecosystems, the planktonic consumers are size-selective feeders [11], [12]. A planktonic food web consists of functional groups, such as meso-zooplankton, rotifers, and ciliates. Thus, the plankton community in lakes and ponds may be useful for examining the factors that control species diversity and community structure across trophic guilds [3].

To test our hypothesis, and reveal the factors determining species diversity and local community structures in natural systems, we investigated variations in species diversity indices and community structures of planktonic groups under a range of local environmental factors. We used a dataset of plankton communities from 18 ponds, and grouped the organisms into 4 taxonomic groups, namely, cladocerans, rotifers, ciliates, and phytoplankton. In addition, we evaluated the whole zooplankton taxa (sum of cladocerans, copepods, rotifers, and ciliates) among the various ecological guilds.

## Materials and Methods

### Study Sites

We conducted our study in 18 ponds in Matsuyama, Japan. All of the ponds were located within an area of 75 km^2^, from 33°48′ N to 33°50′ N, and from 132°48′ E to 132°55′ E, on the plains at the foot of a mountain. The surface areas and water depths ranged from 985 m^2^ to 27,000 m^2^, and from 1.5 m to 7.1 m, respectively. We carried out our field sampling at approximately the center of each pond, during October and November 2005. No specific permits were required for the field studies described, because the location was not privately owned or protected in any way, and the field studies did not involve capturing endangered or protected species.

### Collection and Preparation of Plankton Communities

We collected meso-zooplankton, including cladocerans and copepods, by using vertical net hauls (200-µm mesh). Cladocerans undergo daily vertical migration, and therefore we collected samples from near the bottom to the surface of each pond. For rotifers, we collected water from the surface to a depth of 50 cm, by using a specially designed column sampler, and filtered the collected water through a 40-µm mesh net. All of the collected meso-zooplankton and rotifers were preserved with formalin to a final concentration of approximately 5%. The fixed samples were concentrated to 20 mL, by settling for 24 h. The numbers of individuals of all species were then counted in a 1-mL volume chamber.

To determine the compositions of ciliate and phytoplankton species, we collected 2 L of surface water (depth 0–50 cm) by using the specially designed column sampler. Next, 250-mL portions of each water sample were fixed with acidified Lugol’s solution to a final concentration of 1%, poured into a tapered glass tube, and concentrated to 10 mL by using natural sedimentation. The phytoplankton species were counted in a Burker–Turk hemocytometer under a 400× microscope. For enumeration of ciliates, we fixed and concentrated another 250-mL portion of each water sample as described above. Ciliate cells were identified and counted in a Fuchs–Rosenthal hematocytometer under a 400× microscope.

### Environmental and Biological Factors

To determine the concentrations of chlorophyll *a*, and abundances of heterotrophic nanoflagellates (HNF) and bacteria, we collected 2 L of surface water (depth 0–50 cm) by using the specially designed column sampler. We filtered a 250-mL portion of the sample through a 0.2-µm Nuclepore filter, to retain seston. Next, we placed the filter in a glass test tube and added *N*,*N*-dimethylformamide to extract the chlorophyll *a*, the concentration of which was determined by using a fluorometer (10-AU; Turner Designs, Sunnyvale, USA). For enumeration of bacteria and HNF, additional 100-mL portions of the water sample were fixed with glutaraldehyde to a final concentration of 1%. Bacteria and HNF were counted by using an epifluorescence microscope with ultraviolet excitation (excitation wavelength 330–385 nm), after staining with 4′,6-diamidino-2-phenylindole (DAPI) and primulin, respectively. We measured the Secchi depth at the center of all the ponds, and also the pH of the surface water by using a portable meter (Twin-pH; Horiba Co., Tokyo, Japan). Dissolved organic carbon (DOC) was measured in the sampled surface water, which was filtered through a Whatman GF/F filter, by using a TOC analyzer (TOC-5000; Shimadzu Co., Kyoto, Japan). Total phosphorous (TP) in the surface water was determined by using colorimetric analysis with a continuous flow system (AutoAnalyzer 3; Bran+Luebbe, Norderstedt, Germany).

### Statistical Analyses

For statistical analysis, we used genus as the taxonomic level, because phytoplankton and ciliate identifications were almost always at the genus level. We compared the genus richness among the 4 taxonomic groups (i.e., cladocerans, rotifers, ciliates, and phytoplankton), and also among the whole zooplankton taxa (sum of cladocerans, calanoid copepods, rotifers, and ciliates) by using Pearson’s correlation coefficients (α = 0.05). We used canonical correspondence analysis (CCA) to investigate the relationships between the community structure of each taxonomic group and the environmental factors. CCA is an eigenvalue ordination analysis, which uses the variation in the environmental matrix to explain the variation in the community matrix. For our CCA analyses, we used all of the genera in each taxonomic group and the all environmental factors; all variance inflation factors for these environmental factors were <10, indicating that the co-correlations between the factors statistically influenced the generalized linear model (GLM). We performed permutation tests with 1000 iterations, to test whether the eigenvalues generated by using each CCA were significantly greater than those generated by using a randomized matrix (α = 0.05). To compare the scores of environmental factors among each CCA ordination for each planktonic group, we compared the means of the absolute scores for axes 1–3 among the planktonic groups, and tested the relationships by using Pearson’s correlation coefficients.

We estimated the genus richness and measured the species diversity by using the Shannon–Wiener diversity index, *H*′. We used a GLM to estimate the effects of environmental and biological factors (pH, Secchi depth, TP, DOC, chlorophyll *a,* abundance of bacteria, abundance of HNF, water depth, and pond volume) on the genus richness and *H*′ of each plankton group. All of the environmental factors, except for pH, were log (*x* +1) transformed, based on the results of Shapiro–Wilk normality tests (*a* = 0.05). We used a Poisson distribution for genus richness, and a Gaussian distribution of *H*′ as the error distribution. We selected the best GLM by using downward-stepwise procedure with the Akaike Information Criterion (AIC). All statistics and graphics were performed by using R ver. 2.15.2 software [13].

## Results

**Figure 1:**
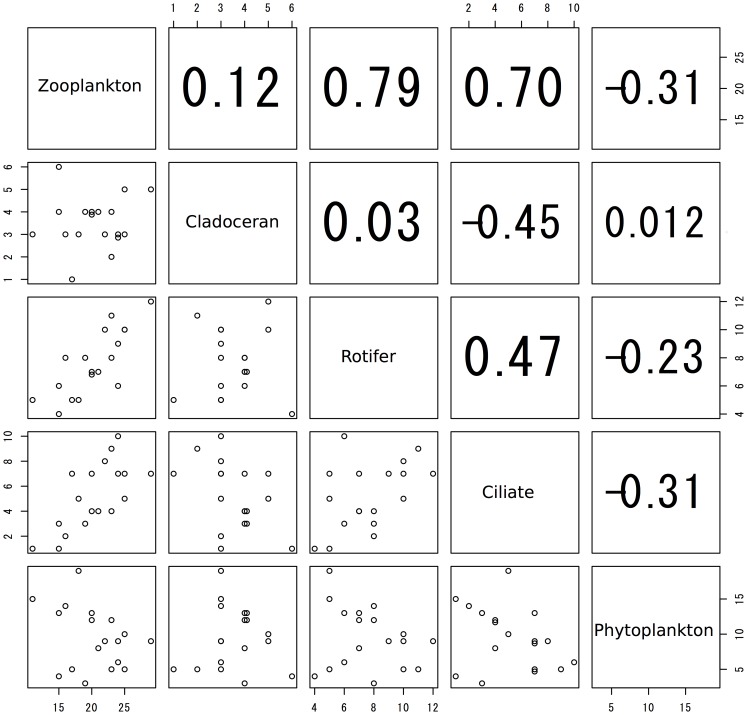
Pair plot for genus richness of plankton taxonomic groups and whole zooplankton. The pair plots showing genus richness of the plankton taxonomic groups, namely, cladocerans, rotifers, ciliates, and phytoplankton, and whole zooplankton. Each point represents a single pond (*n* = 18). The numbers in the upper right-hand columns denote Pearson’s correlation coefficients.

**Figure 2:**
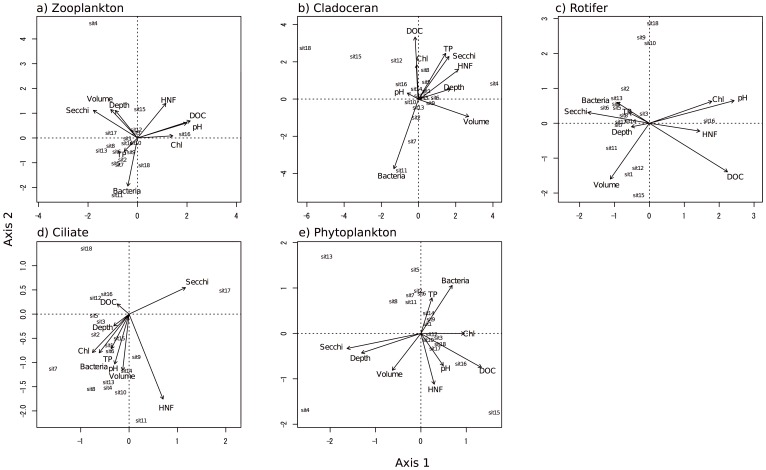
Canonical correspondence analysis results for whole zooplankton and plankton taxonomic groups. Graph showing canonical correspondence analysis results for whole zooplankton (a) and plankton taxonomic groups, namely, cladocerans (b), rotifers (c), ciliates (d), and phytoplankton (e). TP, total phosphorus; DOC, dissolved organic carbon in water; Chl, chlorophyll *a*; HNF, heterotrophic nanoflagellates. Each numbered point indicates an individual site.

**Figure 3:**
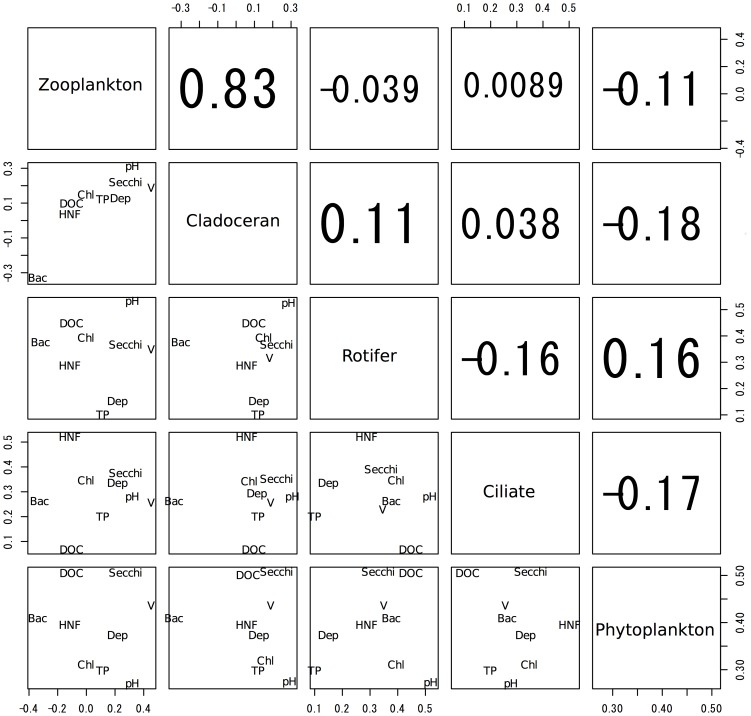
Correlation between planktonic groups based on mean absolute scores of environmental factors. Graph showing the correlation between the planktonic groups based on the absolute scores of the environmental factors used in the canonical correspondence analysis axes 1–3. Each plot represents the absolute score of the environmental factors. TP, total phosphorus; DOC, dissolved organic carbon in water; Chl, chlorophyll *a*; HNF, heterotrophic nanoflagellates; Bac, number of bacteria cells; V, pond volume; Dep, mean water depth. The numbers in the upper right-hand columns denote Pearson’s correlation coefficients. All coefficients were non-significant (*p*>0.05).

For genus richness, significant correlations were observed only between whole zooplankton and ciliates and between whole zooplankton and rotifers (Fig. 1, p<0.01), and not for the others (Fig. 1, p>0.05). For CCA analysis, the total eigenvalues of the CCA axes 1 and 2 ranged from 0.45 to 0.61; therefore, the CCA explained approximately half of the variance through these 2 axes. Further, all of the permutation tests were significant (p<0.05, Fig. 2a–e). In the ordination of whole zooplankton, the CCA revealed that, with the exception of TP, all environmental factors were important in explaining the community composition (Fig. 2a). Meanwhile, the ordination of each planktonic group differed. For cladocerans, all environmental factors except for chlorophyll *a*, pH, and water depth were important (Fig. 2b); for rotifers, DOC, pH, HNF, chlorophyll, Secchi depth, and pond volume were important (Fig. 2c); for ciliates, HNF and Secchi depth were important (Fig. 2d); and for phytoplankton, all environmental factors were equally important (Fig. 2e). The means of the absolute scores for CCA axes 1–3 were not significantly related among any of the planktonic groups, including whole zooplankton (Pearson’s correlation coefficients, p>0.05, Fig. 3). Thus, the planktonic groups responded variously to the environmental factors investigated; further, paired comparison between similar trophic guilds, such as cladocerans and rotifers, showed low correlation.

The GLM results showed that the genus richness and *H*′ of the 4 taxonomic groups and whole zooplankton had various selective factors (Table 1). Genus richness was related to a number of factors; for example, ciliates were significantly related to DOC, while phytoplankton were significantly related to multiple environmental factors. Meanwhile, *H*′ was related to various factors, ranging from water quality to the abundance of microbes. Pond volume was related to the *H*′ of whole zooplankton, rotifers, and phytoplankton, but not to the *H*′ of cladocerans and ciliates. Chlorophyll *a* was a common parameter for determining the diversity of taxonomic groups, and positive relationships were observed between chlorophyll and the parameters Indices of the trophic state, such as Secchi depth and TP, were not strongly related to genus richness or *H*′. Taken together, the GLM results indicate that the genus richness and *H*′ of each taxonomic group were variously influenced by specific environmental factors.

**Table 1:** The best generalized linear model (GLM) results for genus richness and species diversity index (*H*′) of whole zooplankton, and 4 plankton taxonomic groups.

	pH	Secchi depth		TP		DOC		Chl-a		Bacteria		HNF		Depth		Volume		(Intercept)
Genus richness																		
whole zooplankton								0.40***										2.41***
cladocerans																		3.56***
rotifers								0.37								**−**0.28		2.59***
ciliates							1.61*	0.47								**−**0.48		1.77
phytoplankton	−0.23*						−1.32*			−1.01*				−0.25*		0.42		4.86***
H′																		
whole zooplankton								0.269				0.18				−0.29**		1.23**
cladocerans				1.41														0.82***
rotifers				0.31				0.67				−0.7*				0.58**		0.97
ciliates		0					2.05*	0.88*						−0.28**				**−**0.56
phytoplankton	**−**0.25	0*					**−**1.32			**−**0.71						**−**0.36		6.52**

Bold numbers indicate significant factors (p<0.05). No asterisk, p>0.05; *, p<0.05; **, p<0.01; ***, p<0.001. TP, total phosphorus; DOC, dissolved organic carbon in water; Chl-a, chlorophyll *a*; Bacteria, abundance of bacteria; HNF, abundance of heterotrophic nanoflagellates; Depth mean water depth; Volume, pond volume.

## Discussion

In the present study, we have demonstrated variations in genus richness and community structure among the taxonomic groups, according to local environmental factors. Our results indicate that local community responses to environmental factors lack congruence. Contrary to our hypothesis, pairs of similar trophic guilds, such as cladocerans and rotifers, showed low congruence. There are 2 possible explanations for this finding: (1) the existence of niche competition between groups with similar trophic guilds (e.g., cladocerans and rotifers), but not between groups with different trophic guilds (e.g., cladocerans and phytoplankton) and (2) relatively weak effects of trophic interactions on community structure.

Regarding the first explanation, competition may exist between groups with similar trophic guilds under local environmental and biological conditions. In the present study, our analyses tended to show negative relationships between groups with similar trophic positions, except between cladocerans and rotifers, indicating opposite responses of each group to the environmental factors included in our analysis. Indeed, the species of cladocerans, rotifers, and ciliates in the study ponds were complementary [14], and therefore, such niche habitat competition may be responsible for the lack of congruence between groups with similar trophic guilds.

Regarding the second explanation, our study ponds may have been subject to relatively weak effects of trophic interactions on community structure. Some studies have suggested that similarities of richness across trophic levels may be shaped by trophic interactions [15], [16]; moreover, richness at a lower trophic level may have a positive influence on richness at a higher trophic level, because high richness of food resources may allow the co-existence of more species of consumers [17], [18]. Conversely, high species richness of predators may allow more species to co-exist, because some predator species may selectively eliminate prey species that would otherwise tend to dominate the community [19]. However, in the present study, we determined a weak effect of diversity cascades on the plankton community structure. Consumers in aquatic systems may have lesser specializations than those in terrestrial systems [3], [20], [21]. Therefore, although consumers in aquatic systems feed selectively according to the size of their prey species, this size-selective feeding may affect the community structure of the lower trophic level to a lesser extent than in terrestrial systems, where many specialist species exist [3], [8].

The results of the present study revealed some important common factors that can be used to predict the community structures of plankton guilds. One of these factors is pond volume. Allen et al. [5] suggested that most members of a community were related to lake surface area, and that the ecosystem size affected the whole community. Allen et al. [5] collected communities from lakes with 4 orders of lake area; by contrast, our study ponds were within 2 orders of surface area. Therefore, our present results may not be suitable for estimating the effect of ecosystem size in determining a lake community.

Regarding the genus richness and *H*′ index, the factors selected based on the best GLM varied among the planktonic groups. This suggests that species diversity differs among taxonomic groups. Thus, it is difficult to predict the total biodiversity of a system, based upon a single taxonomic group. In other words, a single biodiversity index cannot be used to describe a community within a system [2], [22]–[24]; instead, multiple taxonomic and trophic groups should be used. Our results further suggest that different groups respond variously to environmental fluctuations or change; therefore, 1 trophic group alone should not be used to predict species richness, *H*′, or species composition of another trophic group. Thus, when predicting biodiversity and planning conservation strategies, it is important to consider variations in biodiversity among different taxonomic groups, according to environmental and biological factors.

Our present results strongly concur with those of Longmuir et al. [8], who concluded that individual lake plankton communities respond independently to different environmental factors. In the present study, we also found a weak effect of diversity cascades on the plankton community structure. Thus, in comparison with local environmental factors, trophic interactions would be expected weakly to influence the community structure of each group. Longmuir et al. [8] sampled lakes located in an area of up to 30,000 km^2^, whereas, our study ponds were located within an area of only 75 km^2^. Moreover, Longmuir et al. [8] treated zooplankton, including cladocerans and rotifers, as a single group, whereas we showed that the responses of zooplankton to local environmental factors differed between cladocerans and rotifers. Further studies are required more fully to elucidate the effects of trophic-interaction strength in determining community structure of different trophic guilds.

In the present study, we have demonstrated variations in species diversity among planktonic groups, according to local environmental factors in pond ecosystems. Such a multi-group study provides strong evidence for the patterns of species richness in natural ecosystems. Our study ponds included various trophic states of water quality and pond morphology. Thus, our results highlight the difficulty of predicting biodiversity loss as a result of human impact. When planning conservation strategies to maintain biodiversity in natural ecosystems, it is important to recognize the complexity of variations occurring in species diversity and community structures, according to environmental factors.
